# Cervical Cancer Recurrence and Patient Survival After Radical Hysterectomy Followed by Either Adjuvant Chemotherapy or Adjuvant Radiotherapy With Optional Concurrent Chemotherapy: A Systematic Review and Meta-Analysis

**DOI:** 10.3389/fonc.2022.823064

**Published:** 2022-03-04

**Authors:** Yu-fei Zhang, Yu Fan, Peng Zhang, Jia-ying Ruan, Yi Mu, Jin-ke Li

**Affiliations:** ^1^ Department of Gynaecology and Obstetrics, West China Second Hospital, Sichuan University, Chengdu, China; ^2^ Key Laboratory of Birth Defects and Related Diseases of Women and Children, Sichuan University, Ministry of Education, Chengdu, China

**Keywords:** cervical cancer, radical hysterectomy, radiotherapy, chemoradiotherapy, meta-analysis

## Abstract

**Objective:**

To compare cervical cancer recurrence and patient survival after radical hysterectomy followed by either adjuvant chemotherapy (AC) or adjuvant radiotherapy with or without concurrent chemotherapy (AR/CCRT).

**Methods:**

We systematically searched PubMed, EMBASE, the Cochrane Library and clinicaltrials.gov to identify studies reporting recurrence or survival of cervical cancer patients who received AC or AR/CCRT after radical hysterectomy. Data were meta-analyzed using a random-effects model, and heterogeneity was evaluated using the *I^2^* test. Subgroup and sensitivity analyses were performed to identify potential sources of heterogeneity.

**Results:**

The meta-analysis included 14 non-randomized studies and two randomized controlled trials, altogether involving 5,052 cervical cancer patients. AC and AR/CCRT groups did not differ significantly in rates of total or local recurrence or mortality. Nevertheless, AC was associated with significantly lower risk of distant recurrence [odds ratio (OR) 0.67, 95% confidence interval (CI) 0.55-0.81] and higher rates of overall survival [hazard ratio (HR) 0.69, 95%CI 0.54-0.85] and disease-free survival rate (HR 0.77, 95%CI 0.62-0.92).

**Conclusions:**

AC may be an effective alternative to AR/CCRT for cervical cancer patients after radical hysterectomy, especially younger women who wish to preserve their ovaries and protect them from radiation damage.

**Systematic Review Registration:**

https://www.crd.york.ac.uk/prospero/, identifier PROSPERO (CRD42021252518).

## 1 Introduction

Cervical cancer is the fourth most frequent malignant cancer in women throughout the world, often leading to death (1). In 2020, 604,127 new cases of cervical cancer were reported, leading to approximately 341,831 deaths (2). The apparent incidence of cervical cancer is increasing among younger and premenopausal women, reflecting the greater popularity and availability of cervical screening (3). In China, about 50% of cervical cancer patients are younger than 50 years (2). The prognosis of patients with early-stage cervical cancer is relatively good, and curative surgery can be performed while preserving ovarian function (4).

For patients who have cervical cancer in stages IB-IIA (based on the 2018 FIGO staging system) and do not wish to bear children in the future, the National Comprehensive Cancer Network (NCCN) and the International Federation of Gynecology and Obstetrics (FIGO) recommend radical hysterectomy, bilateral pelvic lymph node dissection and selective oophorectomy (5). Post-surgical adjuvant treatments are recommended for patients with risk factors associated with recurrence or poor survival. So-called “intermediate” risk factors include certain tumor histology (e.g., an adenocarcinoma component), close surgical margins, stromal invasion greater than one third of the cervix, capillary lymphatic space involvement, and cervical tumors with diameters >4 cm (6, 7). “High” risk factors include lymph node metastasis (LNM), parametrial involvement (PMI) and resection margin involvement (RMI) (8). The choice of adjuvant therapy may also depend on other pathological, clinical and surgical factors (9, 10).

About 28-50% of patients with cervical cancer receive postoperative adjuvant therapy (11, 12). The most frequent adjuvant therapies are adjuvant chemotherapy (AC), or adjuvant radiotherapy with or without concurrent chemotherapy (AR/CCRT) (13, 14). Which of these two regimens is better for which types of cervical cancer patients remains unclear. The two therapies have been linked to similar recurrence rates among women with early-stage cervical cancer (15), while other work suggests that AR/CCRT is associated with lower risk of recurrence and morbidity (7). The two regimens have been associated with similar disease-free and overall survival in patients with pelvic lymph node metastases (16). Comparing the two therapies is particularly important in order to decide which may be more suitable for young patients who wish to retain ovary function after radical hysterectomy. For such patients, AC may be better at protecting the ovaries and preserving quality of life (17, 18), but whether the postoperative rates of recurrence and survival are comparable to those after AR/CCRT remains unclear.

To help determine whether AC or AR/CCRT may be preferable for certain types of cervical cancer patient, we performed a systematic review and meta-analysis of the available clinical evidence.

## 2 Methods

This meta-analysis was performed in strict accordance with the Preferred Reporting Items for Systematic Reviews and Meta-analyses (PRISMA) statement. The study protocol was registered in PROSPERO (CRD42021252518).

### 2.1 Search Strategy

The following electronic databases were searched: PubMed, EMBASE, the Cochrane Library and clinicaltrials.gov. We searched all databases from their respective inceptions to February 28, 2021 using the following search strings: [(Cervical Neoplasm) OR (Cervical Cancer) OR (Cervical Tumor) OR (Cervical Carcinoma) OR (Cervix Neoplasm) OR (Cervix Cancer) OR (Cervix Tumor) OR (Cervix Carcinoma)] AND [(Postoperative Therapy) OR (Adjuvant Therapy) OR (Adjuvant Chemotherapy)] AND (Hysterectomy). The reference lists of research articles and reviews were also scrutinized to identify additional studies. In cases of duplicate studies reporting on the same patient population, only the most complete publication was included.

### 2.2 Study Eligibility

We included studies if they reported the following: (1) patients were diagnosed with cervical cancer, and they underwent primary radical hysterectomy involving lymphadenectomy; (2) AC or AR/CCRT was given after radical hysterectomy; (3) relevant outcomes were reported, such as total recurrence, local recurrence, distant recurrence, mortality, overall survival (OS) and disease-free survival (DFS); and (4) the study design was randomized-controlled, observational prospective cohort, retrospective cohort or case-control.

We excluded studies if (1) they did not report original data, e.g., reviews, study protocols, comments or letters; (2) necessary data could not be extracted; (3) they had a single-arm cohort design; (4) they were not published in English; or (5) they failed to score adequately in the quality assessment (see *Study Selection and Quality Assessment*).

### 2.3 Study Selection and Quality Assessment

All literature searches were conducted independently by two reviewers (YF Zhang and Y Fan). After the initial search, duplicate studies were deleted, and the titles and abstracts of the remaining articles were screened to identify potentially eligible studies. Then the reviewers scrutinized the full manuscripts, and those meeting the eligibility criteria were assessed for quality. The quality of non-randomized studies was assessed using the nine-star Newcastle–Ottawa Scale (NOS) (19), with studies earning at least six stars considered “high-quality”. The quality of randomized controlled trials was assessed using the Jadad/Oxford quality scoring system (20), which examines six features: randomization procedure, estimation of sample size, blinding and allocation concealment, loss to follow-up, dropout, and intention-to-treat analysis.

All discrepancies about study selection or quality assessment were resolved through discussion with the corresponding author.

### 2.4 Data Extraction and Calculations of Outcomes

Two reviewers (YF Zhang and Y Fan) independently extracted the following data from each study: name of authors, publication year, study design, sample size, age of patients, FIGO stage, cancer histology, LNM, PMI, RMI, tumor size, deep stromal invasion (DSI), lymphovascular space invasion (LSVI), type of AC, type of adjuvant radiotherapy (AR), recurrence rates (total, local and distant), survival rates (mortality, OS and DFS), and follow-up.

Recurrence was defined as when cervical cancer patients who initially achieved complete remission after primary radical hysterectomy suffered recurrent cancer anywhere in the body, based on histopathology or imaging (21). Local recurrence was defined as recurrence or progression within the pelvis (18) and distant recurrence as recurrence outside the pelvis (18). Recurrence rates were calculated as the number of patients with recurrence, divided by the total number of patients included. Mortality rates were calculated as the number of patients who died of cervical cancer, divided by the total number of patients included. OS and DFS rates were extracted directly from the studies. Any discrepancies were resolved by discussion with the corresponding author.

### 2.5 Statistical Analysis

Meta-analysis was performed using Stata 14.0 (StataCorp, College Station, TX, USA), and results associated with *p* < 0.05 were considered significant. In studies where OS and DFS were reported only as Kaplan-Meier curves, we extracted data using Engauge Digitizer 4.1 (http://sourceforge.net/projects/digitizer/). When appropriate, we calculated pooled odds ratios (ORs) or hazard ratios (HRs) and associated 95% confidence intervals (CIs) using a random-effects model and the DerSimonian-Laird method (22). HR was calculated as described (23).

Heterogeneity of outcomes was assessed based on *I²* and visual analysis of forest plots. We considered *I^2^* >50% as high heterogeneity, in which case we conducted subgroup and sensitivity analyses to obtain more detailed insights and to assess potential sources of heterogeneity (24). Subgroup analyses were based on country, study design, cancer stage, histology, and type of AC or AR. Sensitivity analyses were performed by removing one study at a time and repeating the meta-analysis. Publication bias was assessed using Begg-Mazumdar rank correlation and funnel plots (25).

## 3 Results

### 3.1 Study Selection

**Figure 1:**
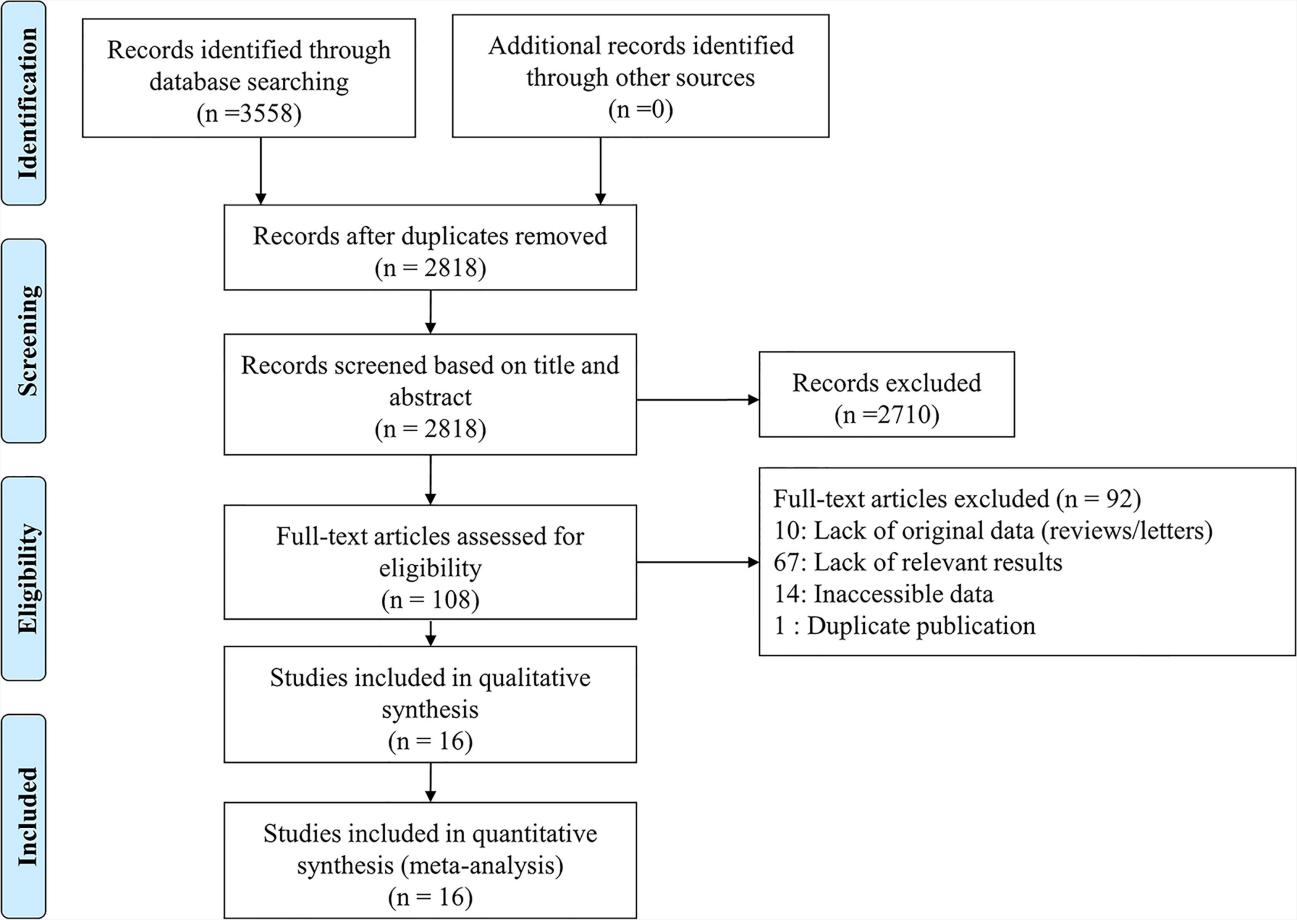
Flow diagram of study selection.

Our search found a total of 3,558 published articles (1,176 in PubMed, 1,888 in Embase, 347 in Cochrane Library and 147 in clinicaltrials.gov). We removed 740 duplicate articles and excluded another 2,710 based on the title or abstract. Full-text review of the remaining 108 articles led to 16 that were included in the systematic review and meta-analysis (26–41). **Figure 1** shows the process of literature selection.

### 3.2 Characteristics of Included Studies

**Table 1** shows the characteristics of the 16 studies, of which 14 were non-randomized and two were randomized-controlled. Altogether the trials involved 5,052 patients with stage IB–IIIB cervical cancer with median ages ranging from 44 to 59 years old who underwent primary radical hysterectomy involving lymphadenectomy. The sample size of each included study ranged from 43 to 2,268 patients. The studies involved the following geographical regions: Japan (n = 7), South Korea (n = 3), China (n = 3), United States (n = 1), Austria (n = 1) and Italy (n = 1). The risk factors in patients that led them to receive adjuvant therapy are listed in **Supplementary Table S1**.

**Table 1:** Characteristics of included studies.

Study	Country	Design	Patients	Median Age (years)	Adjuvant therapy (n)	Regimen	Stage (n)	Histology, n (%)	Median follow-up (months)
Curtin 1996 (26)	USA	RCT	89	45	AC (44)	NTP	IB-IIA (44)	SCC 51 (57.3%), ADC 30 (33.7%), Unknown 8 (9.0%)	36
					AR/CCRT (45)	CCRT	IB-IIA (45)		
Hosaka 2008 (27)	Japan	NRS	70	52.2	AC (28)	NTP	IB (20), IIA (2), IIB (6)	SCC 28 (100.0%)	>36
				50.3	AR/CCRT (42)	RT	IB (22), IIA (1), IIB (19)	SCC 42 (100.0%)	
Hosaka 2012 (28)	Japan	NRS	81	48	AC (32)	TP	IB (17), IIB (15)	SCC 24 (75.0%), ADC/ADSCC 8 (25.0%)	>36
				52	AR/CCRT (49)	RT	IB (21), IIA (3), IIB (25)	SCC 47 (95.9%), ADC/ADSCC 2 (4.1%)	
Iwasaka 1998 (29)	Japan	NRS	180	54.2	AC (53)	NTP	IB (30), IIA (8), IIB (15)	SCC 43 (81.1%), ADSCC 3 (5.7%), Others 7 (13.2%)	75
				52.4	AR/CCRT (127)	RT	IB (73), IIA (18), IIB (36)	SCC 107 (84.3%), ADSCC 7 (5.5%), Others 13 (10.2%)	
Jung 2015 (30)	South Korea	NRS	262	44	AC (85)	TP, NTP	IB (78), IIA (7)	SCC 61 (71.8%), ADC 21 (24.7%), ADSCC 3 (3.5%)	46.8
				48	AR/CCRT (177)	CCRT	IB (152), IIA (25)	SCC 138 (78.0%), ADC 26 (14.7%), ADSCC 13 (7.3%)	
Lahousen 1999 (31)	Austria	RCT	52	51	AC (28)	NTP	IB–IIB (28)	SCC 28 (100.0%)	49
				51	AR/CCRT (24)	RT	IB–IIB (24)	SCC 24 (100.0%)	
Lee 2008 (32)	South Korea	NRS	80	54.5	AC (38)	TP, NTP	IB (32), IIA (6)	SCC 31 (81.6%), ADC 2 (5.3%), ADSCC 5 (13.1%)	49
				56.5	AR/CCRT (42)	RT	IB (37), IIA (5)	SCC 33 (78.6%), ADC 3 (7.1%), ADSCC 6 (14.3%)	
Li 2013 (33)	China	NRS	2268	NR	AC (1010)	TP, NTP	IB-IIA (805), IIB-IIIB (205)	SCC 872 (86.3%), ADC/ADSCC 133 (13.2%), Unknown 5 (0.5%)	41
					AR/CCRT (1258)	RT	IB-IIA (1181), IIB-IIIB (77)	SCC 1214 (96.5%), ADC/ADSCC 41 (3.3%), Unknown 3 (0.2%)	
Li 2016 (34)	China	NRS	133	49	AC (65)	TP	IB (22), IIA (43)	SCC 59 (90.8%), ADC 6 (9.2%)	33.7
				51	AR/CCRT (68)	CCRT	IB (28), IIA (40)	SCC 63 (92.6%), ADC 5 (7.4%)	
Matsuo 2017 (35)	Japan	NRS	1072	47	AC (319)	TP, NTP	IB (202), IIA (34), IIB (83)	SCC 156 (48.9%), ADC/ADSCC 149 (46.7%), Unknown 14 (4.4%)	64.5
				48	AR/CCRT (753)	CCRT, RT	IB (444), IIA (90), IIB (219)	SCC 597 (79.3%), ADC/ADSCC 152 (20.2%), Unknown 4 (0.5%)	
Mossa 2010 (36)	Italy	NRS	263	47	AC (127)	NTP	IB (101), IIA (26)	SCC 127 (100.0%)	120
				49	AR/CCRT (136)	RT	IB (109), IIA (27)	SCC 136 (100.0%)	
Park 2001 (37)	South Korea	NRS	80	45.2	AC (38)	NTP	IB-IIA (38)	SCC 62 (77.5%), ADC 10 (12.5%), Others 8 (10.0%)	52.5
					AR/CCRT (42)	CCRT, RT	IB-IIA (42)		
Seki 2017 (38)	Japan	NRS	135	47	AC (22)	TP, NTP	IB (11), IIA–IIB (11)	ADC/ADSCC 22 (100.0%)	48
				52	AR/CCRT (113)	CCRT, RT	IB (69), IIA–IIB (44)	SCC 90 (79.6%), ADC/ADSCC 23 (20.4%)	
Shen 2019 (39)	China	NRS	43	45	AC (15)	TP	IB-IIA (15)	pure SCCC 31 (72.1%), mixed SCCC 12 (27.9%)	52
				59	AR/CCRT (28)	RT	IB-IIA (28)		
Shimada 2013 (40)	Japan	NRS	133	NR	AC (64)	TP, NTP	IB–IIB (64)	ADC/ADSCC 64 (100.0%)	NR
					AR/CCRT (69)	CCRT, RT	IB–IIB (69)	ADC/ADSCC 69 (100.0%)	
Takekuma 2016 (41)	Japan	NRS	111	45	AC (37)	TP, NTP	IB (23), IIA–IIB (14)	SCC 24 (64.9%), ADC/ADSCC 13 (35.1%)	33
				45	AR/CCRT (74)	CCRT	IB (47), IIA–IIB (27)	SCC 48 (64.9%), ADC/ADSCC 26 (35.1%)	63.3
Total			5052					SCC 4105 (81.3%), ADC 103 (2.0%), Others 844 (16.7%)	

AC, adjuvant chemotherapy; ADC, adenocarcinoma; ADSCC, adenosquamous carcinoma; AR, adjuvant radiotherapy; CCRT, concurrent chemoradiotherapy; NR, not reported; NRS, non-randomized study; NTP, nontaxane and platinum; RCT, randomized controlled trial; RT, radiation therapy; SCC, squamous cell carcinoma; SCCC, small cell carcinoma of the cervix; TP, taxane and platinum.

### 3.3 Quality Assessment of Included Studies

The quality of non-randomized studies was assessed as 7 points (12 studies) or 6 points (two studies; **Table 2**). The quality of the two randomized controlled trials was assessed as 3 (**Table 3**).

**Table 2:** Newcastle-Ottawa scale for assessing risk of bias and quality of non-randomized studies.

Study	Selection				Comparability	Exposure			Total score
	Adequate definition of patient cases	Representativeness of patient cases	Selection of controls	Definition of controls	Control for important or additional factors	Ascertainment of exposure	Same method of ascertainment for participants	Nonresponse rate^*^	
Hosaka 2008 (27)	★	★		★	★★	★	★		7
Hosaka 2012 (28)	★	★		★	★★	★	★		7
Iwasaka 1998 (29)	★	★		★	★	★	★		6
Jung 2015 (30)	★	★		★	★★	★	★		7
Lee 2008 (32)	★	★		★	★	★	★	★	7
Li 2013 (33)	★	★		★	★★	★	★		7
Li 2016 (34)	★	★		★	★	★	★	★	7
Matsuo 2017 (35)	★	★		★	★★	★	★		7
Mossa 2010 (36)	★	★		★	★★	★	★		7
Park 2001 (37)	★	★		★	★	★	★		6
Seki 2017 (38)	★	★		★	★★	★	★		7
Shen 2019 (39)	★	★		★	★★	★	★		7
Shimada 2013 (40)	★	★		★	★★	★	★		7
Takekuma 2016 (41)	★	★		★	★★	★	★		7

One star means one point. A study can be awarded a maximum of one point for each numbered item within the Selection and Outcome categories. A maximum of two points can be given for Comparability.

**Table 3:** Jadad score for assessing risk of bias and quality of randomized controlled trials.

Study	Randomization procedure	Estimation of sample size	Allocation concealment blinding of outcome assessor	Loss to follow-up	Intention to treat analysis	Dropout	Jadad score				
							Randomization	Blinding	An account of all patients		Total score
Curtin 1996 (26)	Yes	Yes	No	Yes	Yes	Yes	2	0	1	3	
Lahousen 1999 (31)	Yes	Yes	No	Yes	Yes	Yes	2	0	1	3	

### 3.4 Recurrence

#### 3.4.1 Total Recurrence Rates

**Figure 2:**
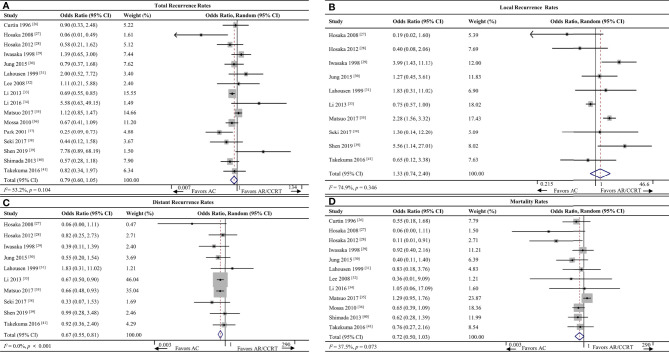
Forest plot of the meta-analysis of **(A)** total recurrence rates, **(B)** local recurrence rates, **(C)** distant recurrence rates or **(D)** mortality rates in AC and AR/CCRT groups. AC, adjuvant chemotherapy; AR, adjuvant radiotherapy; CCRT, concurrent chemoradiotherapy; CI, confidence interval.

Sixteen studies (26–41) including 5,052 patients reported total recurrence rates for the AC group (21.9%, 440/2,005) and the AR/CCRT group (26.9%, 819/3,047). The rates did not differ significantly between the two groups (OR 0.79, 95%CI 0.60-1.05, *p* = 0.104; *I^2^* = 53.2%; **Figure 2A**). Given the high heterogeneity of the pooled data, we conducted subgroup analyses but failed to uncover clear differences among subgroups. Sensitivity analyses identified one study (31) as a potential source of heterogeneity. Excluding this study led to the same result as the full meta-analysis, but with lower heterogeneity (OR 0.75, 95%CI 0.56-1.01, *p* = 0.055; *I²* = 42.3%; **Supplementary Figure S1**).

#### 3.4.2 Local Recurrence Rates

Ten studies (27–31, 33, 35, 38, 39, 41) including 4,274 patients reported local recurrence rates for the AC group (11.0%, 179/1,629) and AR/CCRT group (9.7%, 256/2,645). The rates did not differ significantly between the two groups (OR 1.33, 95%CI 0.74-2.40, *p* = 0.346; *I^2^* = 74.9%; **Figure 2B**). Given the high heterogeneity of the pooled data, we conducted subgroup analyses but failed to uncover clear differences among subgroups. Sensitivity analyses identified one study (29) as a potential source of heterogeneity. Excluding this study led to the same result as the full meta-analysis, but with lower heterogeneity (OR 1.58, 95%CI 0.89-2.80, *p* = 0.121; *I²* = 46.1%; **Supplementary Figure S2**).

#### 3.4.3 Distant Recurrence Rates

Ten studies (27–31, 33, 35, 38, 39, 41) including 4,274 patients reported the distant recurrence rates for the AC group (10.4%, 169/1,629) and AR/CCRT group (16.4%, 435/2,645). AC was associated with a significantly lower rate (OR 0.67, 95%CI 0.55-0.81, *p* < 0.001; *I^2^* = 0.0%; **Figure 2C**).

### 3.5 Survival

#### 3.5.1 Mortality Rates

Twelve studies (26–32, 34–36, 40, 41) including 2,526 patients reported mortality rates for the AC group (17.8%, 164/920) and AR/CCRT group (20.1%, 323/1,606). The rates were similar between the two groups (OR 0.72, 95%CI 0.50-1.03, *p* = 0.073; *I^2^* = 37.5%; **Figure 2D**).

#### 3.5.2 OS Rates

**Figure 3:**
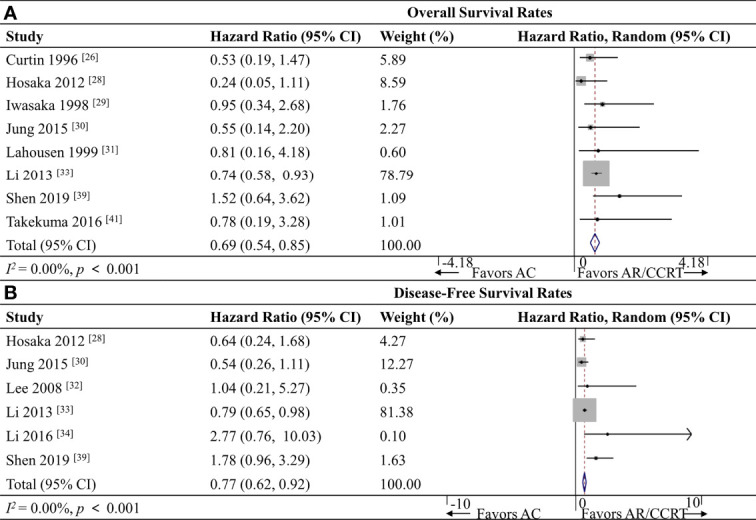
Forest plot of the meta-analysis of **(A)** overall survival rates or **(B)** disease-free survival rates in AC and AR/CCRT groups. AC, adjuvant chemotherapy; AR, adjuvant radiotherapy; CCRT, concurrent chemoradiotherapy; CI, confidence interval.

Eight studies (26, 28–31, 33, 39, 41) including 3,086 patients reported OS data, and meta-analysis associated AC with a significantly better OS rate (HR 0.69, 95%CI 0.54-0.85, *p* < 0.001; *I^2^* = 0.0%; **Figure 3A**).

#### 3.5.3 DFS Rates

Six studies (28, 30, 32–34, 39) including 2,867 patients reported DFS data, and meta-analysis associated AC with a significantly better DFS rate (HR 0.77, 95% CI 0.62-0.92, *p* < 0.001; *I^2^* = 0.0%; **Figure 3B**).

### 3.6 Publication Bias

The Begg-Mazumdar rank correlation test showed no evidence of publication bias in the meta-analysis of recurrence rates (*p* = 0.134), and the funnel plot was symmetrical (**Supplementary Figure S3**).

## 4 Discussion

In this meta-analysis, we evaluated cancer recurrence and survival of patients who underwent radical hysterectomy to treat cervical cancer, followed by AC or AR/CCRT. The two adjuvant therapies were associated with similar risk of total recurrence (OR 0.79, 95%CI 0.60-1.05), local recurrence (OR 1.33, 95%CI 0.74-2.40) and mortality (OR 0.72, 95%CI 0.50-1.03). However, AC was associated with significantly lower risk of distant recurrence (OR 0.67, 95%CI 0.55-0.81) and significantly better OS (HR 0.69, 95%CI 0.54-0.85) and DFS (HR 0.77, 95%CI 0.62-0.92). These findings suggest that AC and AR/CCRT are associated with similar efficacy and, therefore, that AC may be a good alternative for women wishing to retain ovary function after radical hysterectomy.

Our results support the growing use of AC as adjuvant treatment following radical hysterectomy (42, 43), particularly if patients present LNM or advanced cancer (44) or if they wish to retain ovary function. This is increasingly the case as cervical cancer patients are being diagnosed at a younger age (3). The radiation doses in AR/CCRT can damage ovaries permanently, even if ovaries have been transposed (45). The available clinical data suggest that AC is associated with similar prognosis as AR/CCRT, establishing it as an effective and safe alternative, especially for women who want to protect their ovaries.

Nevertheless, our findings should be interpreted with caution because of several limitations. First, our study included a substantial number of patients with cervical cancer in stage IIB and a few patients in stage IIIA or IIIB, whom we could not eliminate from the dataset and who may have influenced our results. FIGO and NCCN guidelines do not recommend radical hysterectomy for these patients. We found no significant difference in OS or DFS between patients in stages IB-IIA (n = 950) and those in stages IB-IIIB (n = 4,102) (data not shown). Second, our meta-analysis pooled data from (a) non-randomized studies, which were larger but may have had greater heterogeneity between AC and AR/CCRT arms; and (b) randomized controlled trials, which were smaller but perhaps had fewer confounding differences between the two arms. Indeed, prevalence of LNM, PMI or RMI were higher in the AR/CCRT group than in the AC group in some studies, which may have confounded comparisons of recurrence and survival. Third, not all studies reported data on all outcomes that we wished to meta-analyze, which may have reduced the statistical power or increased the heterogeneity for certain outcomes. In fact, we observed high heterogeneity in the meta-analyses of total and local recurrence rates, although we were able to identify individual studies contributing substantially to that heterogeneity, and we obtained similar results regardless of whether we omitted those studies. This suggests that even our more heterogeneous meta-analyses are reliable. Fourth, our study did not take into account whether patients underwent minimally invasive surgery or open abdominal surgery. Two meta-analyses concluded that the two types of surgery are associated with similar oncological outcomes (46, 47), but a multi-center, prospective, randomized study linked minimally invasive radical hysterectomy to lower rates of DFS and OS among women with early-stage cervical cancer (11).

In spite of these limitations, our study substantially extends our understanding of the available clinical evidence about outcomes from AC and AR/CCRT. Like the present work, a previous meta-analysis involving 2,663 cervical cancer patients (48) associated AC with lower risk of distant recurrence and similar survival as AR/CCRT. Unlike that meta-analysis, we also compared OS and DFS rates, linking AC to better survival. By updating and expanding the insights into potential differences between AC and AR/CCRT, the present meta-analysis provides a solid basis for considering AC a safe and effective alternative for women who wish to preserve and protect their ovaries following radical hysterectomy.

## Data Availability Statement

The original contributions presented in the study are included in the article/**Supplementary Material**. Further inquiries can be directed to the corresponding author.

## Author Contributions

Y-fZ, conceptualization, data curation, formal analysis, investigation, methodology, writing-original draft. YF, conceptualization, data curation. PZ, data curation, methodology. J-yR, formal analysis, project administration. YM, investigation, methodology. J-kL, project administration, supervision, writing – review & editing. All authors contributed to the article and approved the submitted version.

## Funding

This study was supported by the Science & Technology Department of Sichuan Province, China (2017SZ0118, 2021YJ0124). The funder played no role in the study design; in the collection, analysis or interpretation of data; in the writing of the manuscript; or in the decision to submit it for publication.

## Conflict of Interest

The authors declare that the research was conducted in the absence of any commercial or financial relationships that could be construed as a potential conflict of interest.

## Publisher’s Note

All claims expressed in this article are solely those of the authors and do not necessarily represent those of their affiliated organizations, or those of the publisher, the editors and the reviewers. Any product that may be evaluated in this article, or claim that may be made by its manufacturer, is not guaranteed or endorsed by the publisher.
