# Supplementary figures and images for: Cervical Cancer Recurrence and Patient Survival After Radical Hysterectomy Followed by Either Adjuvant Chemotherapy or Adjuvant Radiotherapy With Optional Concurrent Chemotherapy: A Systematic Review and Meta-Analysis
Source: Front Oncol. 2022 Mar 4;12:823064. doi: 10.3389/fonc.2022.823064 (PMC8931664; doi:10.3389/fonc.2022.823064)

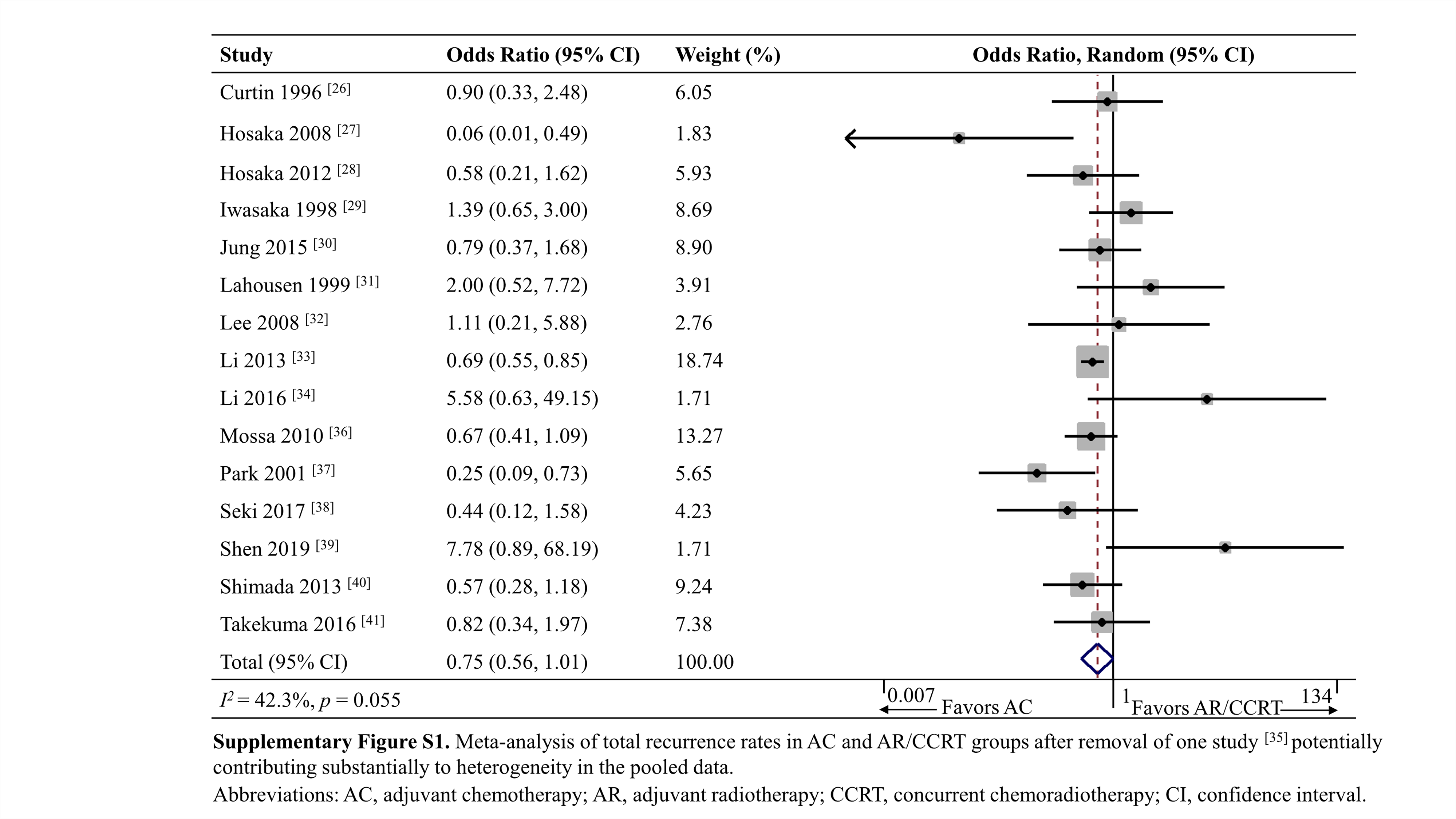

Supplement: Supplementary file 1 [file Image_1.tiff]

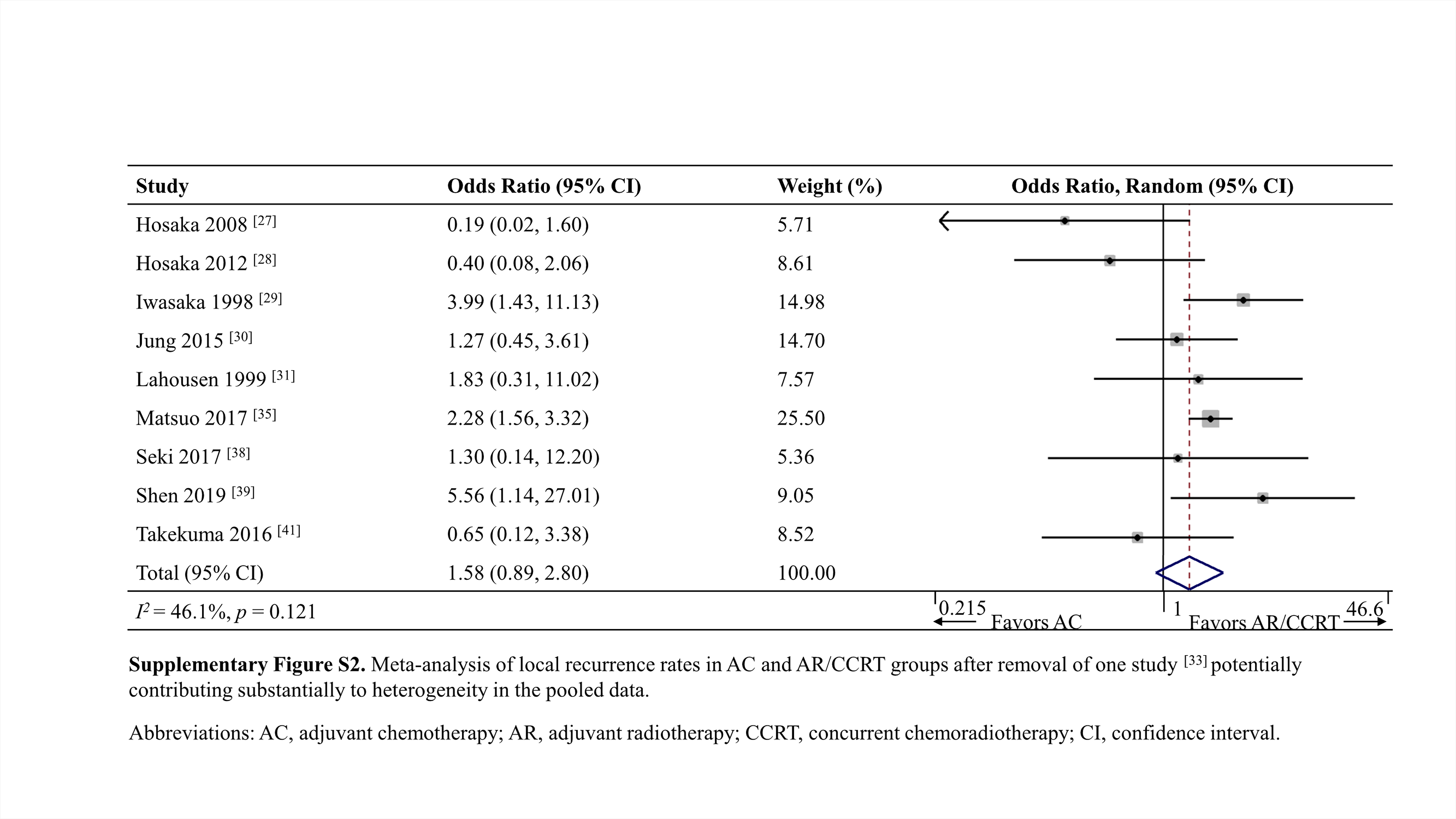

Supplement: Supplementary file 2 [file Image_2.tiff]

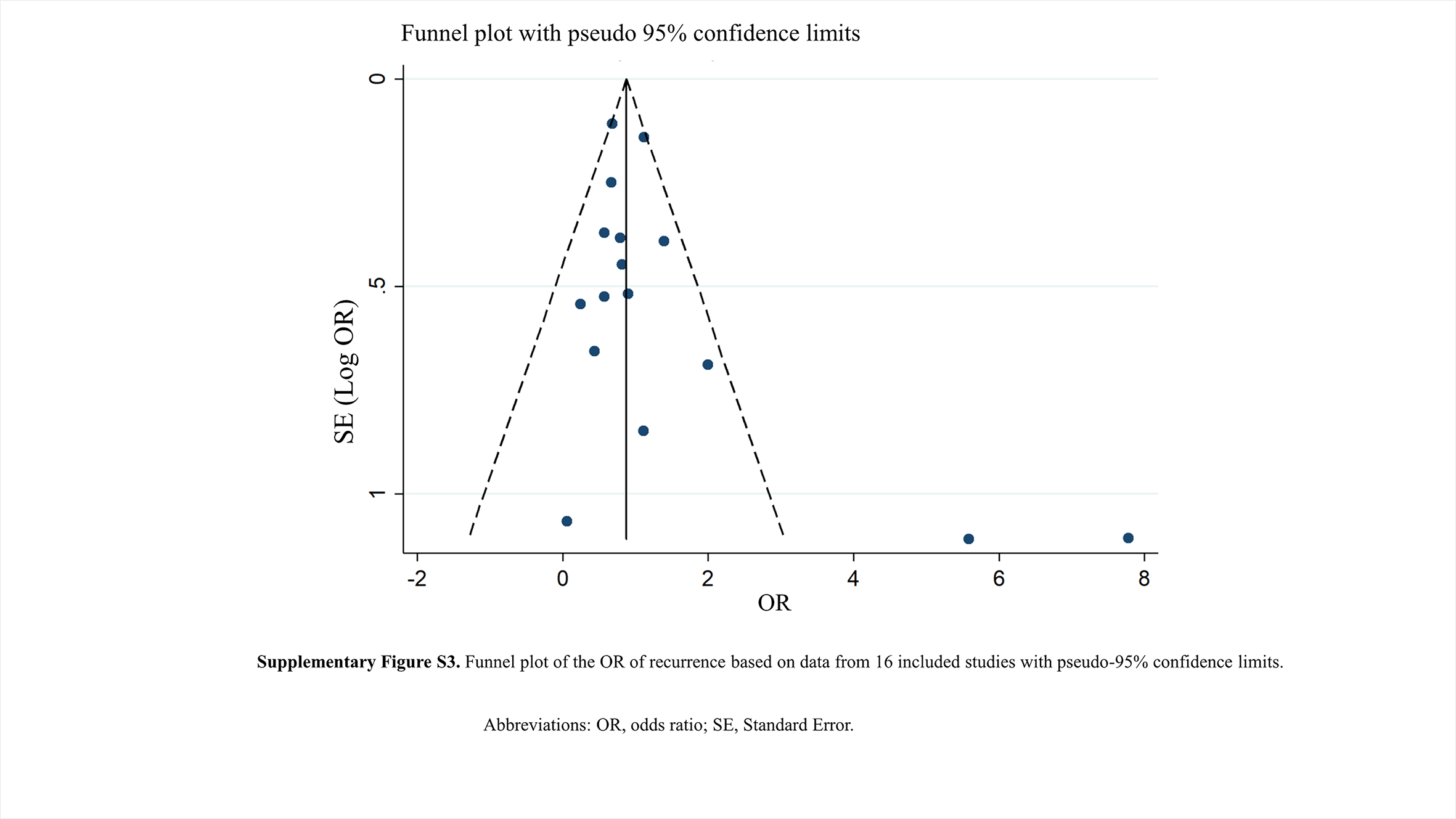

Supplement: Supplementary file 3 [file Image_3.tiff]
