# Supplementary material for: Cervical Cancer Recurrence and Patient Survival After Radical Hysterectomy Followed by Either Adjuvant Chemotherapy or Adjuvant Radiotherapy With Optional Concurrent Chemotherapy: A Systematic Review and Meta-Analysis
Source: Front Oncol. 2022 Mar 4;12:823064. doi: 10.3389/fonc.2022.823064 (PMC8931664; doi:10.3389/fonc.2022.823064)
Supplement: Supplementary file 4 [file Table_1.docx]

**Supplementary Table S1.** Risk factors that led to use of the indicated adjuvant therapies after radical hysterectomy in the studies included in the meta-analysis.

| Study | Group (n) | Intermediate risk factors, n (%) | | |  | High risk factors, n (%) | | |
| --- | --- | --- | --- | --- | --- | --- | --- | --- |
|  |  | Bulky tumor (＞4 cm) | DSI | LSVI |  | LNM | PMI | RMI |
| Curtin 1996 ^[26]^ | AC (44) | 33 (37.1%) | 45 (50.6%) | NR |  | 33 (37.1%) | 9 (10.1%) | NR |
|  | AR/CCRT (45) |  |  | NR |  |  |  | NR |
| Hosaka 2008 ^[27]^ | AC (28) | 10 (35.7%) | 11 (39.3%) | 27 (96.4%) |  | 3 (10.7%) | 5 (17.9%) | 0 (0.0%) |
|  | AR/CCRT (42) | 16 (38.1%) | 24 (57.1%) | 35 (83.3%) |  | 29 (69.0%) | 14 (33.3%) | 0 (0.0%) |
| Hosaka 2012 ^[28]^ | AC (32) | 16 (50.0%) | 19 (59.4%) | 24 (75.0%) |  | 11 (34.4%) | 7 (21.9%) | 0 (0.0%) |
|  | AR/CCRT (49) | 25 (51.0%) | 27 (55.1%) | 40 (81.6%) |  | 22 (44.9%) | 16 (32.6%) | 0 (0.0%) |
| Iwasaka 1998 ^[29]^ | AC (53) | NR | 35 (66.0%) | NR |  | 22 (41.5%) | 16 (30.2%) | 0 (0.0%) |
|  | AR/CCRT (127) | NR | 76 (59.8%) | NR |  | 52 (40.9%) | 40 (31.5%) | 0 (0.0%) |
| Jung 2015 ^[30]^ | AC (85) | NR | 63 (74.1%) | 46 (54.1%) |  | 27 (31.8%) | 8 (9.4%) | 1 (1.2%) |
|  | AR/CCRT (177) | NR | 154 (87.0%) | 99 (55.9%) |  | 80 (45.2%) | 73 (41.2%) | 9 (5.1%) |
| Lahousen 1999 ^[31]^ | AC (28) | NR | NR | 28 (100.0%) |  | 19 (67.9%) | NR | 0 (0.0%) |
|  | AR/CCRT (24) | NR | NR | 24 (100.0%) |  | 14 (58.3%) | NR | 0 (0.0%) |
| Lee 2008 ^[32]^ | AC (38) | 15 (39.5%) | 28 (73.7%) | 17 (44.7%) |  | 0 (0.0%) | 0 (0.0%) | 0 (0.0%) |
|  | AR/CCRT (42) | 20 (47.6%) | 35 (83.3%) | 22 (52.4%) |  | 0 (0.0%) | 0 (0.0%) | 0 (0.0%) |
| Li 2013 ^[33]^ | AC (1010) | 472 (46.7%) | NR | NR |  | NR | NR | NR |
|  | AR/CCRT (1258) | 332 (26.4%) | NR | NR |  | NR | NR | NR |
| Li 2016 ^[34]^ | AC (65) | 0 (0.0%) | 42 (64.6%) | NR |  | 0 (0.0%) | 0 (0.0%) | NR |
|  | AR/CCRT (68) | 0 (0.0%) | 37 (54.4%) | NR |  | 0 (0.0%) | 0 (0.0%) | NR |
| Matsuo 2017 ^[35]^ | AC (319) | 111 (34.8%) | 241 (75.5%) | 270 (84.6%) |  | 319 (100.0%) | 112 (35.1%) | NR |
|  | AR/CCRT (753) | 282 (37.5%) | 531 (70.5%) | 633 (84.1%) |  | 755 (100.0%) | 324 (43.0%) | NR |
| Mossa 2010 ^[36]^ | AC (127) | NR | NR | NR |  | 127 (100.0%) | NR | 0 (0.0%) |
|  | AR/CCRT (136) | NR | NR | NR |  | 136 (100.0%) | NR | 0 (0.0%) |
| Park 2001 ^[37]^ | AC (38) | 3 (3.8%) | NR | NR |  | NR | NR | NR |
|  | AR/CCRT (42) |  | NR | NR |  | NR | NR | NR |
| Seki 2017 ^[38]^ | AC (22) | 17 (77.3%) | NR | NR |  | 8 (36.4%) | 7 (31.8%) | 0 (0.0%) |
|  | AR/CCRT (113) | 73 (64.6%) | NR | NR |  | 66 (58.4%) | 50 (44.2%) | 0 (0.0%) |
| Shen 2019 ^[39]^ | AC (15) | 7 (46.7%) | NR | 11 (73.3%) |  | 9 (60.0%) | NR | NR |
|  | AR/CCRT (28) | 16 (57.1%) | NR | 12 (42.9%) |  | 10 (35.7%) | NR | NR |
| Shimada 2013 ^[40]^ | AC (64) | 26 (40.6%) | 19 (29.7%) | 26 (40.1%) |  | 13 (20.3%) | 12 (18.9%) | 1 (1.6%) |
|  | AR/CCRT (69) | 28 (40.6%) | 17 (24.6%) | 35 (50.7%) |  | 27 (39.1%) | 14 (20.3%) | 7 (10.1%) |
| Takekuma 2016 ^[41]^ | AC (37) | 23 (62.2%) | NR | 34 (91.9%) |  | 29 (78.4%) | 16 (43.2%) | NR |
|  | AR/CCRT (74) | 40 (54.1%) | NR | 67 (90.5%) |  | 62 (83.8%) | 42 (56.8%) | NR |
| Total | AC (2005) | 697/1568 (44.5%) | 458/650 (70.5%) | 483/704 (68.6%) |  | 587/913 (64.3%) | 183/743 (24.6%) | 2/477 (0.4%) |
|  | AR/CCRT (3047) | 832/2462 (33.8%) | 901/1248 (72.2%) | 967/1285 (75.3%) |  | 1253/1704(73.5%) | 573/1516 (37.8%) | 16/770 (2.1%) |

Abbreviations: AC, adjuvant chemotherapy; AR, adjuvant radiotherapy; CCRT, concurrent chemoradiotherapy; DSI, deep stromal invasion; LNM, lymph node metastasis; LVSI, lymphovascular space invasion; NR, not reported; PMI, parametrial invasion; RMI, resection margin involved.
